# Effect of Bioglass on Growth and Biomineralization of SaOS-2 Cells in Hydrogel after 3D Cell Bioprinting

**DOI:** 10.1371/journal.pone.0112497

**Published:** 2014-11-10

**Authors:** Xiaohong Wang, Emad Tolba, Heinz C. Schröder, Meik Neufurth, Qingling Feng, Bärbel Diehl-Seifert, Werner E. G. Müller

**Affiliations:** 1 ERC Advanced Investigator Grant Research Group at the Institute for Physiological Chemistry, University Medical Center of the Johannes Gutenberg University Mainz, Mainz, Germany; 2 Biomaterials Department, Inorganic Chemical Industries Division, National Research Center, Cairo, Egypt; 3 Key Laboratory of Advanced Materials of Ministry of Education of China, School of Materials Science and Engineering, Tsinghua University, Beijing, China; 4 NanotecMARIN GmbH, Mainz, Germany; Université de Lyon - Université Jean Monnet, France

## Abstract

We investigated the effect of bioglass (bioactive glass) on growth and mineralization of bone-related SaOS-2 cells, encapsulated into a printable and biodegradable alginate/gelatine hydrogel. The hydrogel was supplemented either with polyphosphate (polyP), administered as polyP•Ca^2+^-complex, or silica, or as biosilica that had been enzymatically prepared from ortho-silicate by silicatein. These hydrogels, together with SaOS-2 cells, were bioprinted to computer-designed scaffolds. The results revealed that bioglass (nano)particles, with a size of 55 nm and a molar ratio of SiO_2_∶CaO∶P_2_O_5_ of 55∶40∶5, did not affect the growth of the encapsulated cells. If silica, biosilica, or polyP•Ca^2+^-complex is co-added to the cell-containing alginate/gelatin hydrogel the growth behavior of the cells is not changed. Addition of 5 mg/ml of bioglass particles to this hydrogel significantly enhanced the potency of the entrapped SaOS-2 cells to mineralize. If compared with the extent of the cells to form mineral deposits in the absence of bioglass, the cells exposed to bioglass together with 100 µmoles/L polyP•Ca^2+^-complex increased their mineralization activity from 2.1- to 3.9-fold, or with 50 µmoles/L silica from 1.8- to 2.9-fold, or with 50 µmoles/L biosilica from 2.7- to 4.8-fold or with the two components together (100 µmoles/L polyP•Ca^2+^-complex and 50 µmoles/L biosilica) from 4.1- to 6.8-fold. Element analysis by EDX spectrometry of the mineral nodules formed by SaOS-2 revealed an accumulation of O, P, Ca and C, indicating that the mineral deposits contain, besides Ca-phosphate also Ca-carbonate. The results show that bioglass added to alginate/gelatin hydrogel increases the proliferation and mineralization of bioprinted SaOS-2 cells. We conclude that the development of cell-containing scaffolds consisting of a bioprintable, solid and cell-compatible inner matrix surrounded by a printable hard and flexible outer matrix containing bioglass, provide a suitable strategy for the fabrication of morphogenetically active and biodegradable implants.

## Introduction

It is the aim of bone tissue engineering to restore the function of diseased or damaged bone tissue by combining biological and biomechanical laws for the purpose of development of biodegradable scaffolds, allowing isolated functional cells to colonize. In recent years, growing emphasis has been put on three-dimensional (3D) bioprinting of cells to tissue-units due to the various advantages over existing patterning methods especially because it is anticipated that the bioprinting technique allows for the fabrication of personalized, customized free-form implants.

Osseous tissue, bone, is formed by two different structures, cancellous bone and cortical bone. Cancellous bone represents the inner part of bone tissue, is spongy and shows a porosity of 50–90 vol%. In contrast, the dense outer bone layer, the cortical bone, comprises less than 10 vol% porosity. Both bone types undergo a continuous and dynamic remodeling, involving maturation and resorption, that is controlled by the tuned interaction of osteoblasts and osteoclasts, as well as by osteocytes (reviewed in: [1]). Even though bone comprises self-healing ability [2], large-scale bone defects often require external intervention to restore the normal functioning of the skeleton. Besides of using autografts (taken from the same individual) or allografts (from a different donor), bone tissue engineering methods have been introduced to promote and support regeneration of bone or to improve its functions in the patient [3]. The development of functional, biocompatible biomaterials presupposes a thorough understanding of the biology of bone cells and bone tissue formed by them. Both the knowledge of the composition, development and function of the extracellular matrix and the cells of the bone tissue and the growth factors controlling their expression (see: [4]) are pivotal for a successful development of bioactive scaffolds. Very promising are new developments of fabrication of 3D biocompatible structures that can mimic the properties of the extracellular structure, especially with respect to mechanical support, cellular activity and protein production (reviewed in: [5]). Efforts are presently undertaken to bioprint 3D porous bone scaffolds, both by solid free-form fabrication/rapid prototyping [6] and bioprinting of 3D tissue units [7] to create implants that can be replaced, with time, by cells from the donors of the implants.

As hard, bone-imitating scaffold structures, bioglass (bioactive glasses) have been developed (reviewed in: [8], [9]) that turned out to be printable (reviewed in: [10]). Several formulations have been proposed for bioglasses, among which 45S5 Bioglass is the most known one [11], and comprises a molar composition of 46.1 mol.% SiO_2_, 24.4 mol.% Na_2_O, 26.9 mol.% CaO and 2.6 mol.% P_2_O_5_. It forms a strong association with bone [12], comprises osteogenic properties (reviewed in: [9]) and can act as a platform for the formation of organic–inorganic hybrids, e.g. with poly(methyl methacrylate) [13].

The bone cells cannot be embedded into a bioglass scaffold, especially not in a hard porous sintered solid material. However, 3D printed cells, embedded in an aqueous biodegradable material can be placed as tissue units into the bioglass network. Recently we have developed a matrix for bone cells, especially SaOS-2 cells have been used, which contains alginate [14]–[17]. In this matrix, the cells can be readily embedded and bioprinted without losing their proliferation activity. The alginate matrix can be hardened simply by a brief exposure to calcium chloride [15]. In order improve the “biological” environment for the embedded cells low-melding gelatin has been added, since this polymer exposes the highly conserved tripeptide sequence Arg-Gly-Asp which acts as a recognition signal for binding to the cell surface integrin receptors [18]. In addition, we added to the alginate/gelatin hydrogel two further natural polymers, either polyphosphate (polyP) or biosilica. PolyP is synthesized very likely in osteoblasts [19], [20]. Furthermore, polyP displays the potency of SaOS-2 cells and/or mesenchymal stem cells to synthesize mineral deposits and also induces the expression of the key bone enzyme alkaline phosphatase as well as the cytokine bone morphogenetic protein 2 (BMP-2), as well as the major extracellular fibrillar structural molecule collagen type I [21], [22]. For our studies we used polyP together with CaCl_2_ [polyP•Ca^2+^-complex] in order to avoid any depletion of Ca^2+^ ions required for mineral formation [21]. In addition, we added to the hydrogel biosilica, likewise a naturally occurring polymer existing in sponges (phylum: Porifera) where it acts as the inorganic scaffold of the spicules (reviewed in: [4], [23]). Biosilica is formed enzymatically from ortho-silicate by the enzyme silicatein (reviewed in: [23], [24]) and displays an inductive anabolic bone-forming effect on SaOS-2 cells; e.g. this polymer causes a significant shift of the OPG:RANKL (osteoprotegerin:receptor activator of nuclear factor-κB ligand) ratio [25], resulting in an inhibition of the differentiation pathway of pre-osteoclasts into mature osteoclasts. Furthermore, biosilica causes an increased expression of BMP-2 in SaOS-2 cells [25] and shows osteogenic potential [26]. These results have recently been supported using human mesenchymal stem cells [27].

It is the aim of the present study to elucidate if bioglass added to bioprinted SaOS-2 cells from the outside of the alginate/gelatin hydrogel influences the function of the cells by increasing their proliferation and biomineralization properties.

## Material and Methods

### Chemicals

Sodium alginate, phenol red solution [0.5%], gentamycin solution [50 mg/ml], dexamethasone, sodium β-glycerophosphate, ascorbic acid, 3-[4,5-dimethyl thiazole-2-yl]-2,5-diphenyl tetrazolium (MTT), tetraethyl orthosilicate (TEOS), and ammonium hydrogen phosphate were obtained from Sigma-Aldrich (Steinheim; Germany); low-melting gelatin (bovine) from SERVA (Heidelberg; Germany); McCoy's medium and fetal calf serum [FCS] from Biochrom-Seromed (Berlin; Germany); and agarose from Biozym (Hessisch Oldendorf; Germany).

Sodium polyphosphate (Na-polyP of an average chain of 40 phosphate units) was obtained from Chemische Fabrik Budenheim (Budenheim; Germany). The chelating effect, caused by polyP, was compensated by mixing with CaCl_2_ in a stoichiometric ratio of 2∶1 (polyP∶CaCl_2_) as described [21]. This salt was termed “polyP•Ca^2+^-complex”. Usually a concentration of 100 µM (14 µg/ml polyP•Ca^2+^-complex) was added to the assays.

### Hydrogel preparation

A sodium alginate solution (50 mg/ml), supplemented with 50 mg/ml of low-melting gelatin, was prepared in physiological saline. The gel was supplemented with 10 µl/ml of phenol red [0.5%] solution. SaOS-2 cells, growing at the end of the growing phase, were added to the gel at a concentration of 5×10^5^ cells/ml. Where indicated the alginate/gelatin gel was supplemented with 100 µmoles/L polyP•Ca^2+^-complex and 50 µmoles/L ortho-silicate (silica) or 50 µmoles/L biosilica.

Ortho-silicate (silica) was prepared from prehydrolyzed TEOS [28]. Biosilica was prepared enzymatically by using, in a reaction volume of 1 mL, 200 µM prehydrolyzed TEOS and 20 µg/mL of recombinant silicatein in 50 mmoles/L Tris/HCl buffer (pH 7.4; 150 mmoles/L NaCl, 0.5% [w/v] PEG [polyethylene glycol]). PEG was added to the reaction in order to harden the biosilica formed [29], [30]. The enzymatic reactions were performed at 22°C for up to 120 min with agitation. Subsequently the reaction mixture was aspirated and the biosilica content determined by using the molybdate assay; the concentration of the molybdate-reactive silica is given in µmoles/L [31].

### Preparation of bioglass (bioactive glass)

The particles were prepared as described [32], [33]. In order to obtain the bioglass nanoparticles with a molar ratio of Si∶Ca∶P 66∶27∶7, calcium nitrate and TEOS (Sigma) were dispersed in ethanol:water (1∶2 [mol]). The pH of solution was kept to 1.2 with citric acid (Sigma) and the reaction mixture was stirred until the solution became transparent. Then the solution was added at 55°C, under stirring, to distilled water, ammoniated with ammonium hydrogen phosphate (0.4 g/L). The pH value of the reaction solution was kept at ≈11. After a period of stirring overnight the precipitate formed was collected by centrifugation and subsequently washed three times with water. Finally, the white bioglass particles were obtained by separation and finally calcination at 900°C. The particle size was approximately 55 nm, as described [32].

### Three-dimensional cell printing

**Figure 1:**
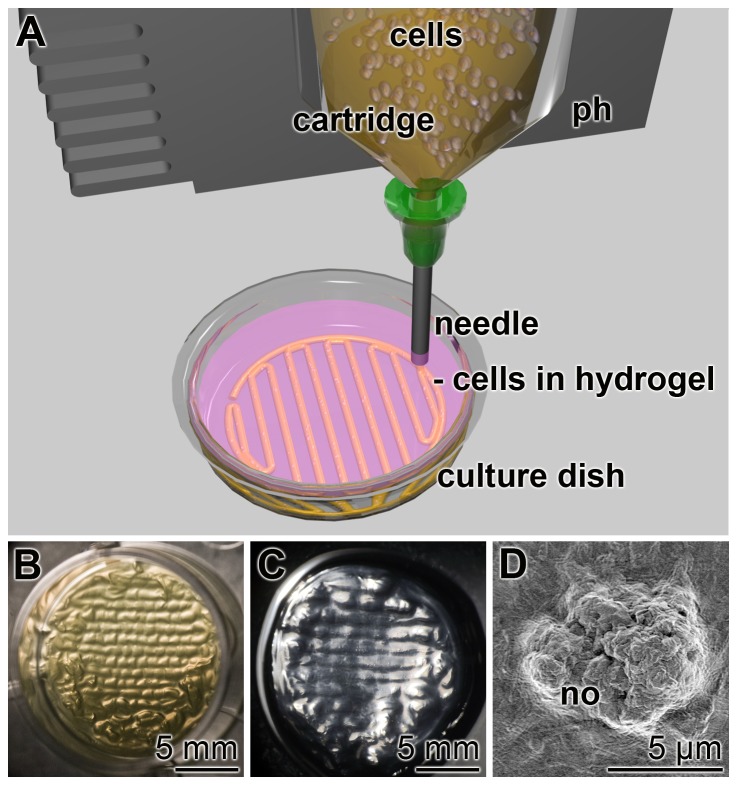
3D cell bioprinting. (**A**) Sketch of the procedure. The alginate/gelatin/SaOS-2 cell suspension is filled into a cartridge, fixed to the printing head (ph). The suspension is pressed through a needle into a culture dish filled with 0.4% CaCl_2_ as cross-linking solution. (**B**) A bioprinted stack of 13 mm in diameter and 1.5 mm in height, placed in a 24-well plate. (**C**) A bioprinted stack after being incubated in medium/FCS for 3 d. (**D**) SEM image of mineral nodules (no) on the surface of SaOS-2 cells embedded in alginate/gelatin and 100 µmoles/L polyP·Ca^2+^-complex and incubated for 3 d in the absence and then for 5 d in the presence of the osteogenic cocktail. The specimen was then inspected by SEM.

The freshly prepared alginate/gelatin/SaOS-2 cell suspension, either as it is, or supplemented with polyP•Ca^2+^-complex, silica or biosilica, was filled into sterile 30 ml printing cartridges (Nordson EFD, Pforzheim; Germany), connected with a 1.98×0.41 mm steel needles (Nordson EFD, Pforzheim; Germany) and placed into the preheated (30°C) printing head of the 3D-Bioplotter (Envisiontec, Gladbeck; Germany). Using the following settings: 0.9 bar pressure, speed of 26 mm/sec, 30°C, the round gel cylinders were printed to a hydrogel scaffold (13 mm diameter; 1.5 mm height). The hydrogel cylinders were injected into Petri dishes, filled with 0.4% CaCl_2_ as cross-linking solution [16]. A sketch is given in Fig. 1A. The cylinders (diameter of 400 µm) were injected in a meander-like pattern by changing the directions of the consecutive layers in a rectangular way. The dimensions of the stacks were computer-controlled using the computer program Bioplotter RP 2.9 CAD software (Envisiontec, Gladbeck, Germany). Finally the hydrogel stacks/scaffolds were transferred with a spatula into 24-well Multiwell Plates (Greiner Bio-One, Frickenhausen; Germany) and submersed in 2.5 mL of McCoy's medium/FCS and incubated at 37°C.

### Cells and their incubation conditions

We used the human osteogenic sarcoma cells, SaOS-2 cells [34] which were cultivated in McCoy's medium containing 2 mmoles/L L-glutamine and gentamycin (50 mg/ml), supplemented with 5% heat-inactivated FCS as described [25]. For some series of experiments the cells were exposed to the osteogenic cocktail (10 nmoles/L dexamethasone, 50 mmoles/L ascorbic acid, 5 mmoles/L sodium β-glycerophosphate) in order to induce biomineralization [25].

Where indicated, the added 2.5 mL of medium/FCS around the bioprinted cylinders, formed of alginate/gelatin/SaOS-2 cells without or with polyP•Ca^2+^-complex, silica or biosilica, were supplemented with 5 mg/ml of bioglass (nano)particles. For the determination of the effects on growth incubation lasted 3 d. In order to determine the effect of the different components on the mineralization capacity of SaOS-2 cells the cultures were incubated for 3 d in the absence of the osteogenic cocktail and subsequently for 5 d in the presence of the cocktail.

### Microscopic analyses

The scanning electron microscopic (SEM) images were performed with a HITACHI SU 8000 (Hitachi High-Technologies Europe GmbH, Krefeld; Germany) at low voltage (<1 kV; analysis of near-surface organic surfaces) detector. The SEM mapping experiments (energy dispersive spectrometry [EDS] at sub-micrometer spatial resolution) were performed with a XFlash FlatQUAD (Bruker Nano, Berlin; Germany) using the following settings, 5.5 kV, 260 pA, 120 kcps, 640×480 pixels, 33 nm pixels, 62 min.

Digital light microscopic studies were performed using a VHX-600 Digital Microscope (Keyence, Neu-Isenburg; Germany) equipped with a VH-Z25 zoom lens.

### Cell viability and proliferation

The polymers/hydrogels had been tested in the assays as follows: Control assays without any component (termed “none”). Additional components in the hydrogel samples that had been used for printing had been (*i*) ortho-silicate, prepared from prehydrolyzed TEOS (“silica”), (*ii*) biosilica, prepared enzymatically from prehydrolyzed TEOS and silicatein (“biosilica”), (*iii*) silicatein alone without any silica precursor (“silicatein”), (*iv*) polyP•Ca^2+^-complex (“polyP”) and finally (*v*) polyP•Ca^2+^-complex together with biosilica (“polyP + biosilica”). The assays with these printed hydrogel samples had been performed in the absence (“minus bioglass”) or presence of bioglass, added to the medium/serum (“plus bioglass”).

The MTT cell viability assay was applied to determine the cell concentration on the basis of the activities of the dehydrogenase enzymes. After 3 d in culture/incubation the numbers of SaOS-2 cells were determined after having treated the alginate/gelatin hydrogel with 55 mmoles/L Na-citrate as described [16], [35]. Subsequently, the cell suspension was incubated with fresh medium containing 100 µl of MTT for 12 h in the dark. After removal of the remaining MTT dye 200 µl of dimethylsulfoxide (DMSO) were added to solubilize the formazan crystals. Finally the optical densities (OD) at 570 nm were measured using an ELISA reader/spectrophotometer [36], with background subtraction at 630–690 nm. Ten parallel experiments were performed.

### Mineralization by SaOS-2 cells in vitro

Like described for the cell viability assays the respective assays of the hydrogels, composed for the mineralization studies have been termed “none”, hydrogel without any component; “silica”, addition of ortho-silicate, prepared from TEOS; “biosilica”, prepared from prehydrolyzed TEOS and silicatein; “silicatein”, addition of only the enzyme; “polyP”, addition of polyP•Ca^2+^-complex; or finally “polyP + biosilica”, polyP•Ca^2+^-complex together with biosilica. Those hydrogel samples, containing the cells, had been printed and then incubated in medium/serum in the absence (“minus bioglass”) or presence of bioglass (“plus bioglass”).

The mineral deposits onto the cells were stained with Alizarin Red S. The cells were incubated for 3 d in the absence of the osteogenic cocktail and subsequently for 5 d in the presence of the cocktail. Then the hydrogel was gently dissolved by addition of 55 mmoles/L Na-citrate and the cells were allowed to adhere to the plastic bottom of the 24-well plates during a 24 h incubation period. After a further careful washing cycle with physiological saline (without sudden shaking) the reaction with Alizarin Red S staining reaction was performed. The color developed was quantitatively assessed, using the fluorochrome Alizarin Red S as an indicator by applying the spectrophotometric assay [25], [37]. Briefly, SaOS-2 cells were immersed in acetic acid, mechanically removed from the plastic bottom and pelleted. The supernatant obtained was neutralized with ammonium hydroxide and the optical density was read at 405 nm. The moles of bound Alizarin Red S were determined after obtaining of a calibration curve. The amount of bound Alizarin Red S is given in µmoles. Values were normalized to total DNA in the samples. The DNA content was determined using the PicoGreen method [38], with calf thymus DNA as a standard.

### Statistical analysis

The results were statistically evaluated using paired Student's *t*-test [39] and the two-way ANOVA test [40]. The generation time (number of cell doublings per given incubation period [3 d]) of SaOS-2 cells was calculated according to Powell [41].

## Results and Discussion

Bioglass is a well established hard, porous basis material for the formation of bone-replacing scaffolds [8]–[11].

### Preparation of the bioglass

**Figure 2:**
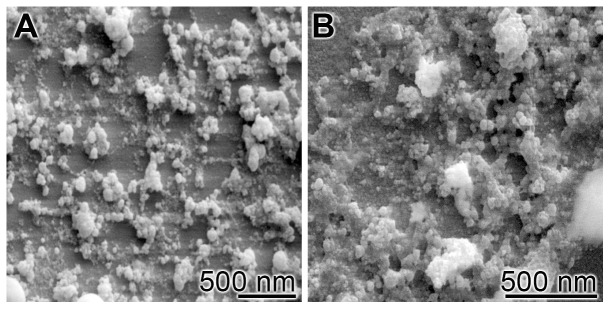
Morphology of the bioglass preparation used. (**A** and **B**) SEM micrograph. The sintering temperature was 900°C.

As solid (nano)particles of bioglass we used the solid glass powder, prepared according to and characterized by Hong *et al*. [32]. Using the procedure described under “Material and Methods”, the size of the particles was determined to be 55 nm (Fig. 2). The molar ratio of the elements SiO_2_∶CaO∶P_2_O_5_ was 55∶40∶5 [33]. By this, the composition of the bioglass is high in phosphorus compared to the one of 45S5 and Bioglass described by Hench [11], with 46 mol% SiO_2_, 24 mol% Na_2_O, and 30 mol% CaO.

### Bioprinting of *SaOS-2* cells in alginate/gelatin

The SaOS-2 cells were encapsulated into a hydrogel with the components alginate and gelatin, as described under “Material and Methods”. The bioprinted cylinders were approximately 300 µm thick and the parallel oriented patterned layers were arranged in a perpendicular orientation in the consecutive layers (Fig. 1B and C).

**Figure 3:**
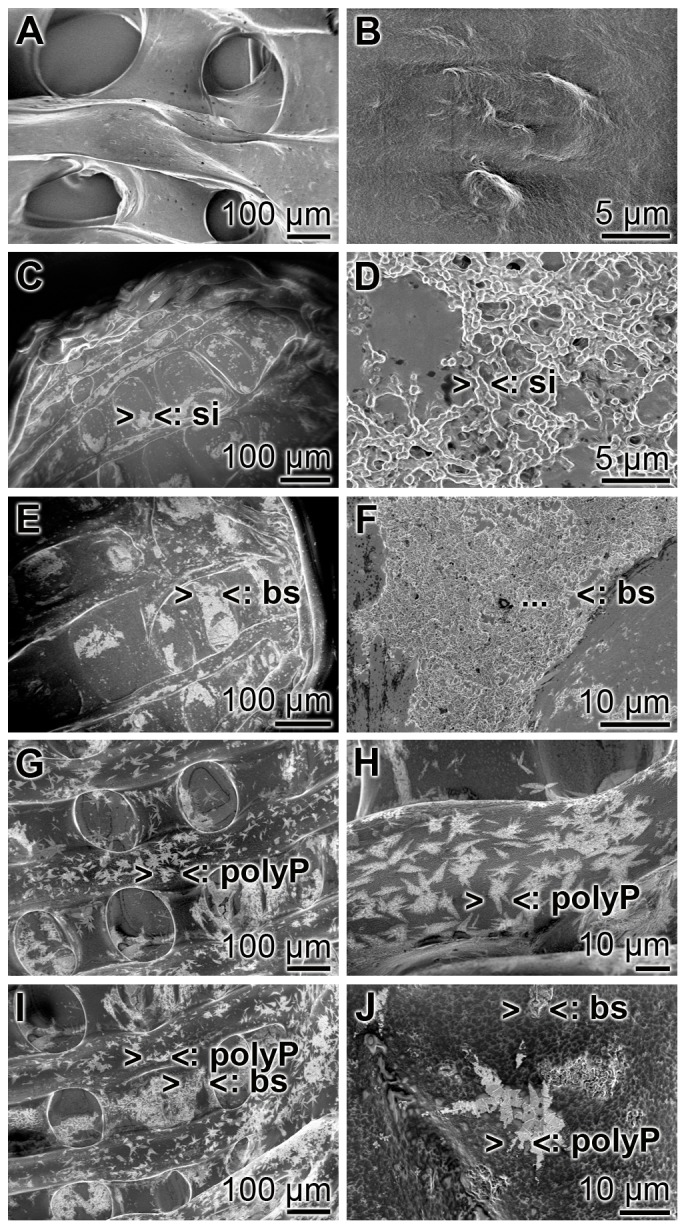
SEM images of cell-free printed alginate/gelatin hydrogels. (**A**) Appearance of the basic alginate/gelatin hydrogel cylinders, containing neither silica nor biosilica nor polyP·Ca^2+^-complex. (**B**) Hydrogel containing 10 µg/ml of silicatein. (**C** and **D**) Printed basic alginate/gelatin hydrogel containing 50 µmoles/L ortho-silicate (>si<). (**E** and **F**) Cylinders of hydrogel supplemented with 50 µmoles/L biosilica (>bs<). (**G** and **H**) Alginate/gelatin hydrogel cylinders containing 100 µmoles/L polyP·Ca^2+^-complex (>polyP<). (**I** and **J**) Hydrogel containing both 50 µmoles/L biosilica (>bs<) and 100 µmoles/L polyP·Ca^2+^-complex.

SEM analyses revealed that the samples containing only alginate/gelatin show an almost homogeneous appearance (Fig. 3A). Addition of 10 µg/ml of silicatein to the gel does not change the homogeneous pattern (Fig. 3B). However, if 50 µmoles/L ortho-silicate (final concentration) is added to the hydrogel the samples comprise clusters of 500 nm-sized silica drops (Fig. 3C and D). The clusters and patches of biosilica (50 µmoles/L) within the hydrogel are widespread (Fig. 3E and F). Addition of 100 µmoles/L polyP•Ca^2+^-complex to the hydrogel, prior to the bioprinting process, revealed the appearance of crystal-like precipitates in the hydrogel cylinders (Fig. 3G and H). The size of those deposits range between 10 and 15 µm. If the hydrogel is supplemented both with polyP•Ca^2+^-complex and biosilica the characteristic deposits, crystal-like for polyP•Ca^2+^-complex and round-shaped clusters of drops for biosilica become obvious (Fig. 3I and J).

### Effect of polyP, silica and biosilica on growth of SaOS-2 cells in the alginate/gelatin hydrogel

**Figure 4:**
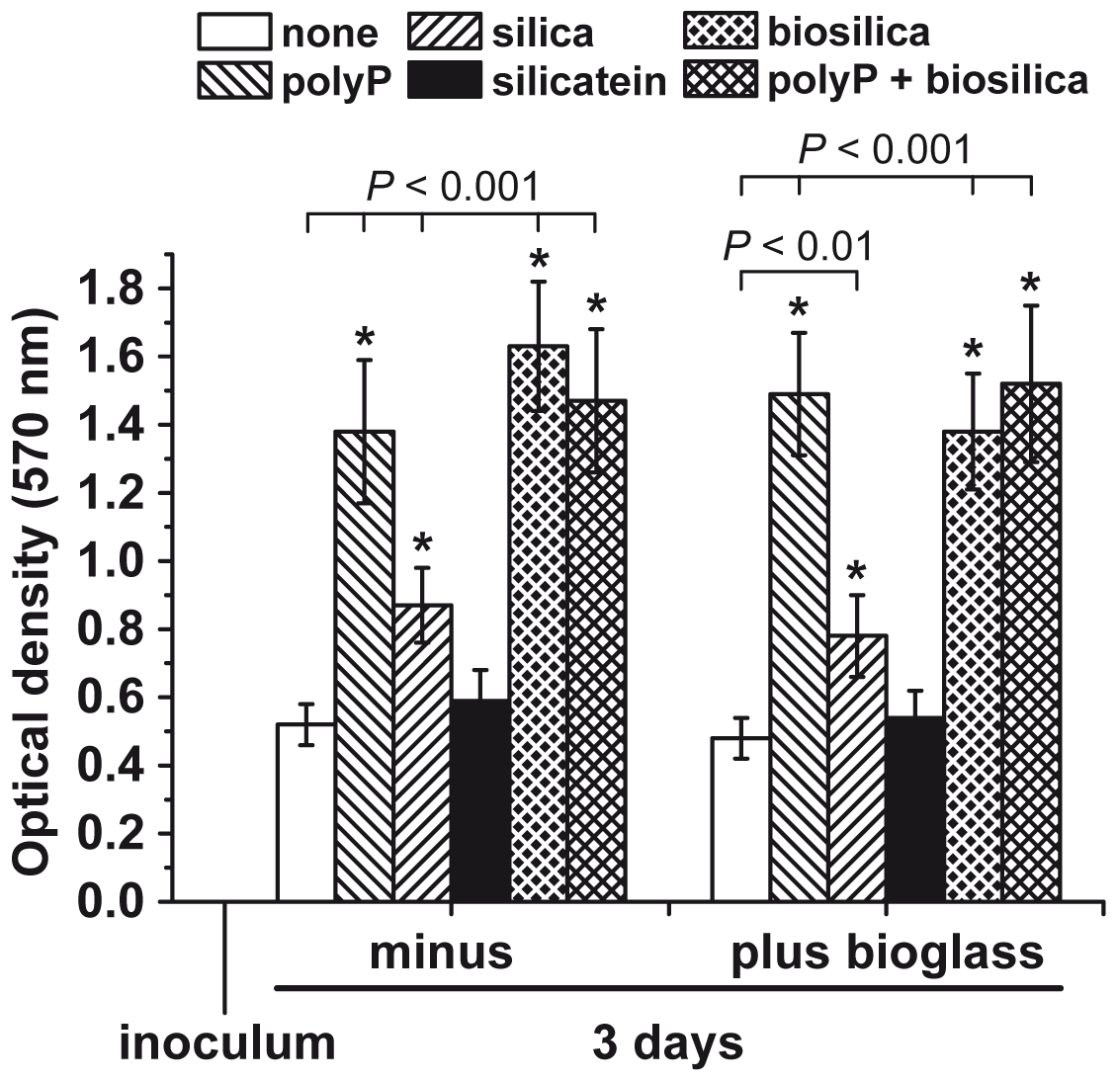
Influence of the polymers silica (50 µmoles/L; hatched rightwards bars), biosilica enzymatically prepared from ortho-silicate and silicatein (50 µmoles/L; cross hatched white on black), polyP•Ca^2+^-complex (polyP) (100 µmoles/L; hatched leftwards), and polyP•Ca^2+^-complex together with biosilica (polyP + biosilica) (cross hatched black on white) on cell density after an incubation period of 3 d in medium/FCS as determined by MTT assay . The values for the optical density reflect the overall activity of the mitochondrial dehydrogenases and are correlated with the cell number. Control experiments, either containing no polymer (open bars) or silicatein (filled bars), were performed in parallel. The experiments were performed either in the absence (minus bioglass) or presence of 5 mg/ml of bioglass (nano)particles (plus bioglass) in culture medium/FCS. Data represent means ± SD of ten independent experiments, each. Significant differences between the controls (no polymer added) and a second set of data, from one experimental group (with either polyP, silica, biosilica, or polyP + biosilica), in the absence or presence of bioglass, had been calculated. The respective values for the significance (Student's *t*-test) are marked with * *P*<0.01 or *P*<0.001, as highlighted in the figure. In addition the results had been analyzed by the two-way ANOVA test (see “Results”).

In the absence of the bioglass in the medium the two natural polymers, polyP•Ca^2+^-complex and biosilica, but also to a smaller extent ortho-silica, display a significant stimulation of cell growth, if the cell density is measured after an incubation period of 3 d (Fig. 4). Addition of 100 µmoles/L polyP•Ca^2+^-complex caused a significant (calculated between the respective experimental set and the control [without any of the polymers]) increase in cell density from 0.52±0.06 optical density units (controls without any of those polymers) to 1.38±0.21 units. Addition of 50 µmoles/L biosilica to the hydrogel resulted in an increase to 1.63±0.22 units; co-addition of 100 µmoles/L polyP•Ca^2+^-complex and 50 µmoles/L biosilica did not change the cell density significantly. This might imply that under these conditions the maximal potency of the cells to proliferate has been reached. During the 3 d incubation period the cells underwent 1.6 doublings (1.6-fold increase in cell density during the 3 d incubation). If 50 µmoles/L of chemically polycondensed silica, as ortho-silicate, is added to the hydrogel the cell growth increased to a smaller extent, but still significantly, to 0.87±0.11 units. In contrast, 10 µg/ml of silicatein added alone without ortho-silicate substrate to the hydrogel, had no significant effect on the cell density (Fig. 4). However, if silicatein (10 µg/ml) was added together with 50 µmoles/L ortho-silicate to the alginate/gelatin hydrogel the SaOS-2 cells proliferated and reached a cell density of 1.48±0.27 units (data not included in Fig. 4). This result indicates that also in the hydrogel environment silicatein, if added together with its substrate ortho-silicate, has the capacity to form enzymatically biosilica.

A statistical analysis of the values for the cell growth in the absence and presence of bioglass had been performed by two-way analysis of variance (ANOVA). The analyses revealed that all experimental test series between the two groups, without and with bioglass, with the exception of the assays that contained no additional component (termed "none" in Fig. 4) or contained silicatein ("silicatein" in Fig. 4), like silica, biosilica, polyP•Ca^2+^-complex as well as polyP•Ca^2+^together with biosilica, are significantly different (*P*<0.001). Significantly higher values for the optical density units had been measured for those experimental sets which contained bioglass.

Addition of 5 mg/ml of bioglass (nano)particles to the cells embedded into alginate/gelatin hydrogel, containing in separate series of experiments, the polymers polyP•Ca^2+^-complex, silica, biosilica or polyP•Ca^2+^-complex together with biosilica did not significantly change the growth potency of the cells (Fig. 4); the extent of the cell densities was very similar to those seen in the assays lacking bioglass.

### Influence of the polymers on biomineralization

**Figure 5:**
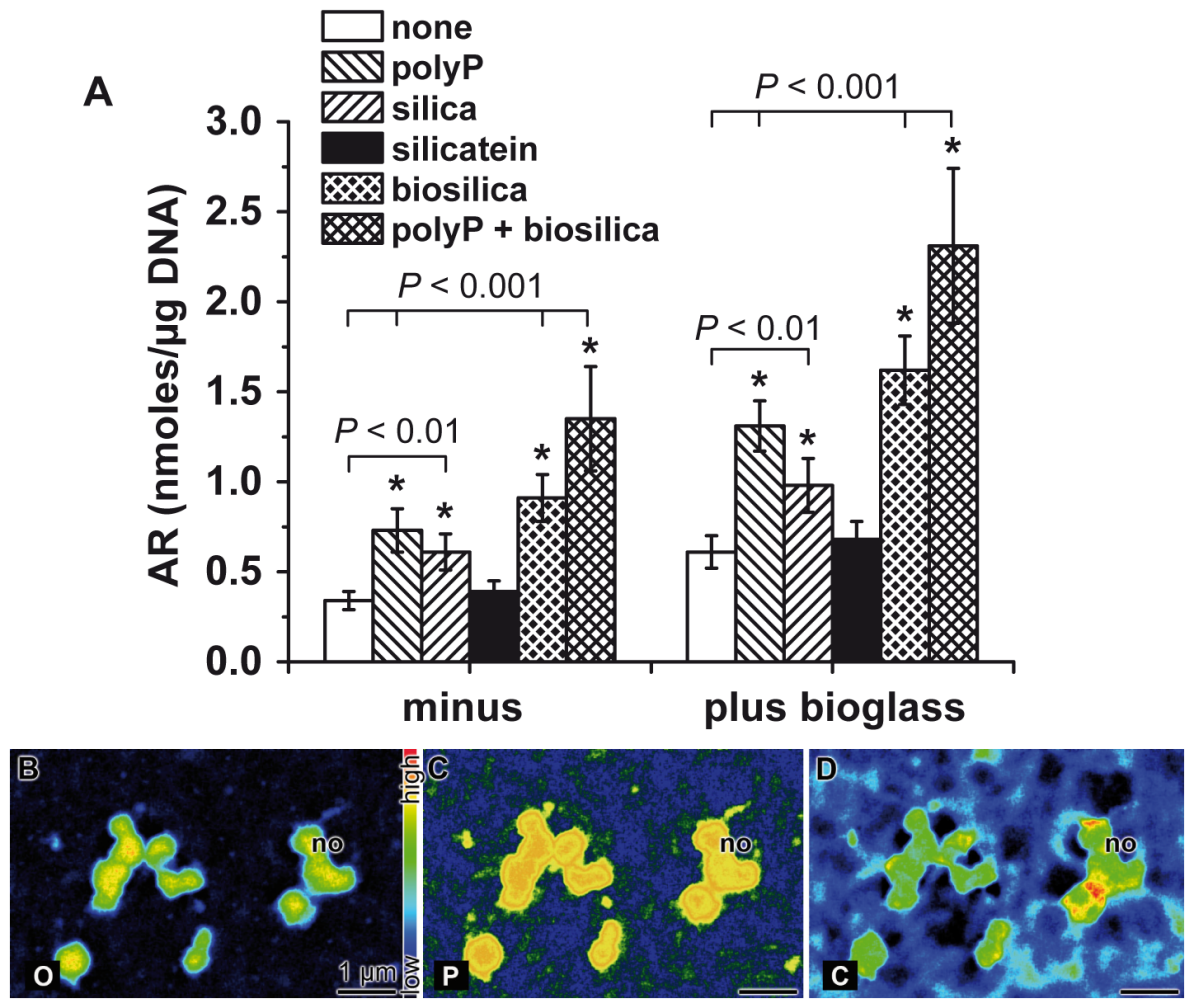
Formation of minerals onto SaOS-2 cells. (**A**) Influence of the tested polymers, polyP·Ca^2+^-complex (polyP) (100 µmoles/L), silica (50 µmoles/L), biosilica (50 µmoles/L), and polyP·Ca^2+^-complex together with biosilica (polyP + biosilica) on the extent of biomineralization, measured by binding of Alizarin Red S to the inorganic deposits. The color reaction was followed spectroscopically at 570 nm; the values are correlated with the DNA content in the respective sample to allow a direct correlation with the cell numbers. The assays were run in the absence (minus) or presence of 5 mg/ml of bioglass (plus bioglass) in the incubation medium for the organic, printed scaffold. The results shown come from eight experiments; the means ± SD are indicated. Significance between the values from the controls (no polymer added) and one series of a given experimental set, in the absence or presence of bioglass, had been calculated and is marked, either with * *P*<0.01 or * *P*<0.001 (Student's *t*-test). The statistical evaluation by two-way ANOVA test is given under “Results”. (**B** to **D**) Element mapping by EDS of the surface of SaOS-2 cells comprising mineral nodules. One nodule is marked (no). Element mapping was performed for O (B), P (C) and C (D). The regions of brighter pseudocolor represent larger accumulations of the respective elements.

As outlined under “Material and Methods” the SaOS-2 cells embedded into alginate/gelatin hydrogel, supplemented with the different polymers examined here, were incubated for 3 d in the absence of the osteogenic cocktail and then for additional 5 d in the presence of the cocktail. The extent of biomineral formation was determined spectrophotometrically and the values were correlated with the DNA amount in the respective sample. By that, a correlation of the magnitude of mineral deposits on the SaOS-2 cells could be quantified. As summarized in Fig. 5A the amount of Alizarin Red S-positive reaction, in the assay without added bioglass, increased significantly from 0.34±0.05 nmoles of Alizarin Red S formed/µg DNA to 0.73±0.12 nmoles/µg (for 100 µmoles/L polyP•Ca^2+^-complex), to 0.61±0.10 nmoles/µg (50 µmoles/L silica), to 0.91±0.13 nmoles/µg (50 µmoles/L biosilica) and to 1.38±0.29 nmoles/µg (100 µmoles/L polyP•Ca^2+^-complex together with 50 µmoles/L biosilica); the values in the controls without any polymer and with 10 µg/ml silicatein vary around 0.35 nmoles/µg. Addition of 5 mg/ml of bioglass to the incubation assay increased the extent of mineralization significantly, for polyP•Ca^2+^-complex to 1.31±0.14 nmoles/µg, for silica to 0.98±0.14 nmoles/µg, for biosilica to 1.62±0.19 nmoles/µg and for the two components together (polyP•Ca^2+^-complex and biosilica) to 2.31±0.43 nmoles/µg (Fig. 5). Likewise, also in the absence of any additional polymer the potency of the embedded SaOS-2 increased significantly from 0.34±0.05 nmoles/µg DNA, in the absence of bioglass in the culture medium, to 0.61±0.09 nmoles/µg in the presence of bioglass.

Again, these data had been statistically evaluated by two-way ANOVA. A statistically significant increase (*P*<0.001) is seen between the samples lacking bioglass and those that had been treated with bioglass (Fig. 5), with the exception for the assays that had not been supplemented with silica, biosilica, polyP•Ca^2+^-complex as well as polyP•Ca^2+^together with biosilica, and the exception of silicatein.

To ascertain that only the minerals, synthesized by the cells had been quantified by the Alizarin Red S staining procedure, the scaffold had been printed without cells. After an incubation period of 5 d the hydrogel was dissolved by addition of 55 mmoles/L Na-citrate. After a washing step with saline and a subsequent keeping of the well plates in the culture chamber, the staining reaction was performed. In all assays, irrespectively if initially the hydrogel contained polyP, biosilica or silica the absorbance at 405 nm was determined. The values obtained had been lower than 0.8 nmoles dye bound per 1 cm^2^ of the plastic bottom (data not shown here).

### Crystallite formation onto SaOS-2 cells

Incubation period of 5 d of the cells in medium/FCS, supplemented with osteogenic cocktail, results in the formation of mineral deposits. Those 1 to 10 µm large deposits can be visualized by SEM (Fig. 1 D).

In order to make sure that the crystallites, formed onto the SaOS-2 cells indeed contain phosphate we analyzed the samples, after SEM analysis by energy-dispersive X-ray spectroscopy. In turn, the EDS analysis hat been performed at sub-micrometer spatial resolution; the iamages revealed an accumulation of the signals for the elements O, P and C (Fig. 5B-D); the element Ca is likewise present at higher concentrations at the mineral nodules (not shown). This finding indicates that the deposits are composed of Ca-phosphate, but also of considerable high amounts of Ca-carbonate. The latter finding confirms recent observations that newly growing mineral deposits contain besides of Ca-phosphate also Ca-carbonate [42].

## Conclusions

The free-formed fabrication of bone implants requires the solution of two printable matrices. First a solid, bendable porous matrix and a more soft cell biocompatible hydrogel into which the embedded cells can proliferate and differentiate. In the present study it is shown that the bone-related SaOS-2 cells can be embedded into an alginate/gelatin matrix which, after supplementation with silica, but especially with polyP and biosilica, allows the cells to proliferate. Based on earlier findings that both polyP and biosilica display the potency to enhance the induction of morphogenetically active cytokines, e.g. BMP-2 [4], it can be assumed that the embedded and bioprinted cells also differentiate. The organic hydrogel scaffold is composed of cylinders which leave room not only for the outgrowth of the cells for (hopefully) initiating vascularization but also for additional scaffold materials that can likewise be printed. Among those is bioglass that has been proven its beneficial role in bone implants and can be printed [10], also for organic polymers, e.g. chitin and chitosan. Both polymers have been shown to resist to bioprinting [43], [44]. At the present time we are working on the a reinforcement of the bioprinted material by adding (poly-)cations to the scaffold. Following this line of research to develop a solid and concurrently flexible and integrable printable hard scaffold which surrounds a more solid and cell compatible inner matrix that is likewise bioprintable, we hope to contribute further to a progress in the technology to fabricate bioreplacable implants.
